# Extraction of Anthraquinones from Japanese Knotweed Rhizomes and Their Analyses by High Performance Thin-Layer Chromatography and Mass Spectrometry

**DOI:** 10.3390/plants9121753

**Published:** 2020-12-11

**Authors:** Vesna Glavnik, Irena Vovk

**Affiliations:** Department of Food Chemistry, National Institute of Chemistry, Hajdrihova 19, SI-1000 Ljubljana, Slovenia; vesna.glavnik@ki.si

**Keywords:** Japanese knotweed, *Reynoutria*, *Polygonum*, *Polygonaceae*, anthraquinones, emodin, physcion, HPTLC, HPTLC-MS, densitometry

## Abstract

Anthraquinones (yellow dyes) were extracted from Japanese knotweed rhizomes with twelve extraction solvents (water; ethanol_(aq)_ (20%, 40%, 60%, 70% and 80%), ethanol, 70% methanol_(aq)_, methanol, 70% acetone_(aq)_, acetone and dichloromethane). The obtained sample test solutions (STSs) were analyzed using high-performance thin-layer chromatography (HPTLC) coupled to densitometry and mass spectrometry (HPTLC–MS/MS) on HPTLC silica gel plates. Identical qualitative densitometric profiles (with anthraquinone aglycones and glycosylated anthraquinones) were obtained for STSs in all the solvents except for the STS in dichloromethane, which enabled the most selective extractions of anthraquinone aglycones emodin and physcion. The highest extraction efficiency, evaluated by comparison of the total peak areas in the densitograms of all STSs scanned at 442 nm, was achieved for 70% acetone_(aq)_. In STS prepared with 70% acetone_(aq)_, the separation of non-glycosylated and glycosylated anthraquinones was achieved with developing solvents toluene–acetone–formic acid (6:6:1, 3:6:1 and 3:3:1 *v*/*v*) and dichloromethane–acetone–formic acid (1:1:0.1, *v*/*v*). Non-glycosylated anthraquinones were separated only with toluene–acetone–formic acid, among which the best resolution between emodin and physcion gave the ratio 6:6:1 (*v*/*v*). This solvent and dichloromethane–acetone–formic acid (1:1:0.1, *v*/*v*) enabled the best separation of glycosylated anthraquinones. Four HPTLC-MS/MS methods enabled the identification of emodin and tentative identification of its three glycosylated analogs (emodin-8-*O*-hexoside, emodin-*O*-acetyl-hexoside and emodin-*O*-malonyl-hexoside), while only the HPTLC-MS/MS method with toluene-acetone-formic acid (6:6:1, *v*/*v*) enabled the identification of physcion. Changes of the shapes and the absorption maxima (bathochromic shifts) in the absorption spectra after post-chromatographic derivatization provided additional proof for the detection of physcion and rejection of the presence of chrysophanol in STS.

## 1. Introduction

Japanese knotweed (*Fallopia japonica* Houtt., *Polygonaceae*; synonyms: *Polygonum cuspidatum*, *Polygonum reynoutria* and *Reynoutria japonica*) is on the list of the “100 World’s Worst Invasive Alien Species”, because it represents huge ecological (biodiversity loss) and economic problems (damage of infrastructure) in Europe and North America. However, in the environment of its origin, which is Eastern Asia, it is used in traditional Chinese and Japanese medicine for healing infections, inflammatory diseases, hyperlipidemia and other diseases [1]. Traditional applications (powder, extracts and herbal infusions) are usually prepared from rhizomes (subterranean stems). Rhizomes contain many secondary metabolites, including stilbenes (especially *trans*-resveratrol and its glycosylated analogs [2,3,4,5,6,7,8,9,10,11]); proanthocyanidins (from monomers [2,6,8,12,13], dimers [2,6,8,12,13], oligomers [2,4,8,12,13] and up to polymers [2,8,12,13]); phenolic acids [2,6,8]; phenylpropanoid glycosides [2,4]; flavonoids [2,8]; naphthalenes [2,3,4,6]; triterpenoids [8] and anthraquinones (especially emodin [2,3,5,6,7,9,10,11,14], physcion [2,3,5,6,7,9,10,11,14] and their glycosides [2,3,4,6,9,11,14] and other anthraquinones [2,4,6,7,12]). Leaves of Japanese knotweed rhizomes have been shown to be rich in proanthocyanidins [15] and carotenoids [16].

Anthraquinones are the largest group of natural dyes, with about 700 compounds [17], and can be found in many plant genera, such as *Cassia* [18,19], *Aloe* [17], *Rheum* [20] and *Fallopia* [2], to which Japanese knotweed belongs. Anthraquinones present in Japanese knotweed are traditionally used as laxatives. Besides laxative activities [21], anthraquinones show antibacterial [1,10], antiviral [1,21], antifungal [1], anticancer [22] and estrogenic [1] properties. They also showed a potential to be skin-whitening agents, as they act as tyrosinase inhibitors [9]. Emodin showed antibacterial activities against foodborne bacteria [10] and antifungal activities against phytopathogenic fungi [23]. Physcion has the potential to be an anticancer agent for the treatment of nasopharyngeal carcinoma [22]. Emodin and its glycosides were chosen as marker compounds in Chinese pharmacopoeia [4].

Analyses of anthraquinones, extracted from Japanese knotweed rhizomes with boiling water [6], pure ethanol [9], methanol [3,4,11], aqueous ethanol [7,24,25,26] and aqueous methanol [5,10], were performed with methods based on high-performance liquid chromatography (HPLC with a fluorescence detector (HPLC-FLD) [9], photo-diode array detector (HPLC-PDA) [24] and (U)HPLC-PDA-MS^n^ [2,3,4,5,6,7,8,10,11]) and thin-layer chromatography (TLC) [25,26]. TLC methods for anthraquinones from Japanese knotweed rhizomes were used for the quantification of emodin [25], for the isolation of fractions [26] and for the testing of Japanese knotweed rhizomes fermentation products [27]. TLC methods for analyses of anthraquinones in other plant materials were applied for the screening [28,29] and quantification of anthraquinones [18,19,30,31,32]. TLC was also applied for analyzing the mycelium and culture supernatant rich in anthraquinones [29] Separations were mainly performed on a silica gel stationary phase (TLC silica gel plates [19,25,29], TLC silica gel plates F_254_ [28,30,31,33] and TLC silica gel F_254_ G plates [18]) in combination with the following developing solvent mixtures: ethanol–water [28], petroleum ether–ethyl acetate–formic acid [26], toluene–ethyl acetate [19], hexane–ethyl acetate [18], ethyl acetate–methanol–water [30], toluene–ethyl formate–formic acid [33], petroleum ether–ethyl formate–formic acid [29], petroleum–butyl acetate–methanol–acetic acid [25] and toluene–ethyl acetate–formic acid [31]. Additionally, TLC RP-18 F_254_ plates were developed with methanol–water–formic acid [32].

The aims of the study were: (i) selection of extraction solvents for the extraction of anthraquinones from the Japanese knotweed rhizomes, (ii) selection of developing solvents for the separation of non-glycosylated and glycosylated anthraquinones and (iii) development of the first high-performance thin-layer chromatography-mass spectrometry (HPTLC-MS/MS) methods for the separation and identification of anthraquinones from Japanese knotweed rhizomes.

## 2. Results and Discussion

### 2.1. Selection of Extraction Solvent

**Figure 1:**
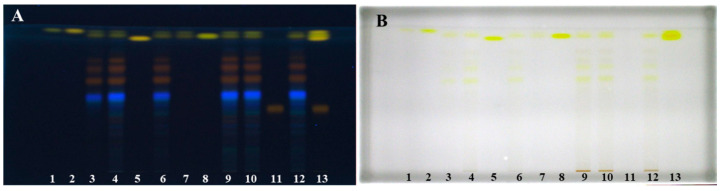
HPTLC chromatograms of physcion (1 μg; track 1), chrysophanol (1 μg; track 2), aloe-emodin (1 μg; track 5), emodin (1 μg; track 8) and aloin A (1 μg; track 11) standards and Japanese knotweed rhizomes STSs (2 μL, 50 mg/mL) prepared with acetone (track 3), methanol (track 4), ethanol (track 6), dichloromethane (track 7), 70% acetone_(aq)_ (track 9), 70% ethanol_(aq)_ (track 10) and 70% methanol_(aq)_ (track 12). HPTLC silica gel plates were developed with toluene–acetone–formic acid (3:6:1, *v*/*v*) and documented after development at 366 nm (**A**) and at white light (**B**).

Optimization of extraction of anthraquinones (yellow dyes) and their glycosylated derivatives from the rhizomes of the Japanese knotweed was performed on an analytical scale. Different extraction solvents like water, 20% ethanol_(aq)_, 40% ethanol_(aq)_, 60% ethanol_(aq)_, 70% ethanol_(aq)_, 80% ethanol_(aq)_, ethanol, 70% methanol_(aq)_, methanol, 70% acetone_(aq)_, acetone and dichloromethane were used for the preparation of separate sample test solutions (STSs) from rhizomes. STSs were analyzed on two separate HPTLC silica gel plates. STSs prepared with 70% ethanol_(aq)_, ethanol, 70% methanol_(aq)_, methanol, 70% acetone_(aq)_, acetone and dichloromethane were on the first plate together with standards of physcion, chrysophanol, aloe-emodin, emodin and aloin A (Figure 1). 

**Figure 2:**
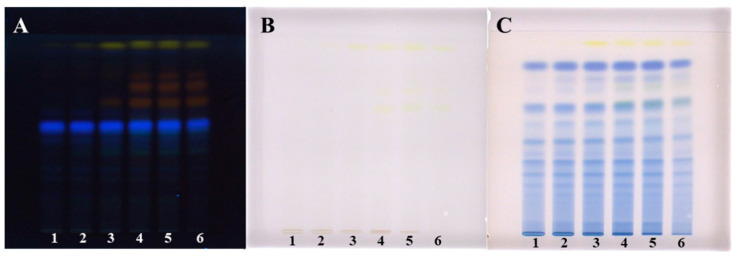
HPTLC chromatograms of Japanese knotweed rhizomes STSs (2 μL, 50 mg/mL) prepared with water (track 1), 20% ethanol_(aq)_ (track 2), 40% ethanol_(aq)_ (track 3), 60% ethanol_(aq)_ (track 4), 80% ethanol_(aq)_ (track 5) and ethanol (track 6). HPTLC silica gel plates were developed with toluene–acetone–formic acid (3:6:1, *v*/*v*). Images of the plates were documented at 366 nm (**A**) and at white light (**B**) after development and at white light (**C**) after derivatization with 4-dimethylaminocinnamaldehyde (DMACA) reagent.

On the second plate were STSs prepared with 20% ethanol_(aq)_, 40% ethanol_(aq)_, 60% ethanol_(aq)_, 80% ethanol_(aq)_ and ethanol (Figure 2). Both plates were developed with the developing solvent toluene–acetone–formic acid (3:6:1, *v*/*v*). As shown in Figure 1 (tracks 1, 2, 5, 8 and 11), all standards were detected at 366 nm and at white light illumination conditions. At 366 nm standards of physcion, chrysophanol, aloe-emodin and emodin were detected as yellow bands at R_F_ values in the range of 0.88–0.93, while standard of aloin A (track 11) was detected as orange-brown band at R_F_ 0.42. At white light, all standards appeared as yellow bands (Figure 1B). At 366 nm, two yellow bands were detected at R_F_ 0.90 and 0.93 in tracks of STSs prepared with ethanol, 70% ethanol_(aq)_, methanol, 70% methanol_(aq)_, acetone, 70% acetone_(aq)_ and dichloromethane (Figure 1A, tracks 3, 4, 6, 7, 9, 10 and 12). Similarity between the colors of the band of aloin A standard (Figure 1, track 11) and the bands of STSs prepared with ethanol, 70% ethanol_(aq)_, methanol, 70% methanol_(aq)_, acetone and 70% acetone_(aq)_ at R_F_ values 0.60, 0.69 and 0.74 (Figure 1, tracks 3, 4, 6, 9, 10 and 12) was observed. These bands that appear orange-brown at 366 nm (Figure 1A) are yellow at white light (Figure 1B). Two intensive yellow bands at R_F_s 0.90 and 0.93 were detected at 366 nm and at white light on the second HPTLC plate but only for STSs prepared with 60% ethanol_(aq)_, 80% ethanol_(aq)_ and ethanol (Figure 2, tracks 4–6). The yellow band at R_F_ 0.90 with comparable intensity was observed also for STSs prepared with 40% ethanol_(aq)_, while a much lower intensity was detected for STSs prepared with water and 20% ethanol_(aq)_ (Figure 2, tracks 1–3). At 366 nm, three bands with the same orange-brown color as was observed for aloin A standard (Figure 1, track 11) were detected for STSs prepared with 60% ethanol_(aq)_, 80% ethanol_(aq)_ and ethanol (Figure 2, tracks 4–6) at R_F_ values 0.60, 0.69 and 0.74. These bands were yellow at white light.

**Figure 3:**
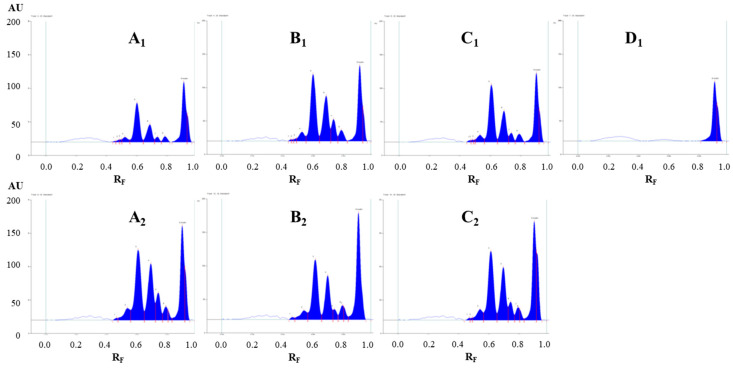
Densitograms of Japanese knotweed rhizomes STSs (2 μL, 50 mg/mL) scanned at 442 nm in absorption/reflectance mode. STSs (2 μL, 50 mg/mL) were prepared with acetone (**A_1_**), 70% acetone_(aq)_ (**A_2_**), methanol (**B_1_**), 70% methanol_(aq)_ (**B_2_**), ethanol (**C_1_**), 70% ethanol_(aq)_ (**C_2_**) and dichloromethane (**D_1_**). HPTLC silica gel plate was developed with toluene–acetone–formic acid (3:6:1, *v*/*v*).

**Figure 4:**
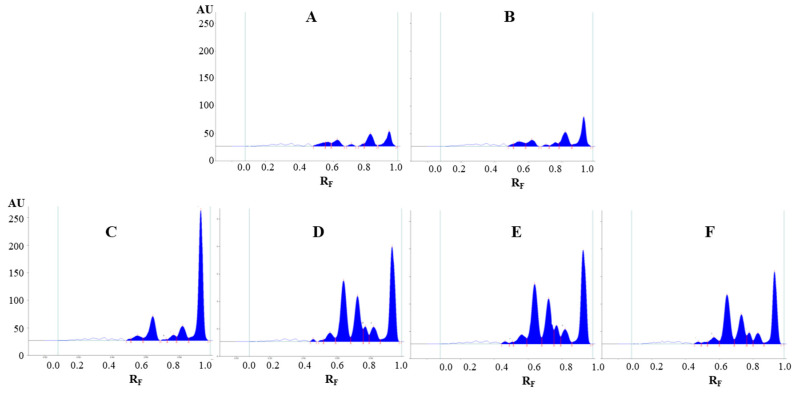
The densitograms of Japanese knotweed rhizomes STSs (2 μL, 50 mg/mL) scanned at 442 nm in absorption/reflectance mode. STSs (2 μL, 50 mg/mL) were prepared with water (**A**), 20% ethanol_(aq)_ (B), 40% ethanol_(aq)_ (**C**), 60% ethanol(_aq_) (**D**), 80% ethanol_(aq)_ (**E**) and ethanol (**F**). HPTLC silica gel plate was developed with toluene–acetone–formic acid (3:6:1, *v*/*v*).

Evaluation of the chromatograms obtained by different extraction solvents on both plates was performed based on densitometric scanning at 442 nm in absorption/reflectance mode (Figure 3 and Figure 4). The wavelength 442 nm (absorption maximum of emodin, known in the literature to be present in Japanese knotweed rhizomes) was selected based on the absorption spectra of all standards scanned in situ on the developed HPTLC silica gel plate. The absorption maxima for physcion, chrysophanol, aloe–emodin, emodin and aloin A were at 442, 428, 442, 360 and 431 nm, respectively.

The densitograms scanned at 442 nm for STSs prepared with different pure organic solvents (acetone, methanol, ethanol and dichloromethane) and mixtures of organic solvents with water (70% acetone_(aq)_, 70% methanol_(aq)_ and 70% ethanol_(aq)_) show similarities and differences in the number and intensities of the peaks (Figure 3). Based on these observations, it can be concluded that dichloromethane is a more selective extraction solvent than all other tested solvents, as no peaks were detected in the densitogram below the R_F_ value 0.8 (Figure 3D_1_). All other extraction solvents show equal qualitative profiles in the densitograms (Figure 3A_1_–C_1_). The use of extraction solvents that contained water (70% acetone_(aq)_, 70% methanol_(aq)_ and 70% ethanol_(aq)_) resulted in higher peaks compared to pure organic solvents (acetone, methanol and ethanol). Comparison of the densitograms for STSs, prepared with water, 20% ethanol_(aq)_, 40% ethanol_(aq)_, 60% ethanol_(aq)_, 80% ethanol_(aq)_ and ethanol, showed equal qualitative profiles (Figure 4). The increasing of the % of ethanol (from 20% to 80%) in the extraction solvent results in a significant increase in all heights of all the peaks (Figure 4B–E). In the case of STS prepared with pure ethanol, the peak heights were lower than for STS prepared with 80% ethanol_(aq)_ (Figure 4E,F).

**Figure 5:**
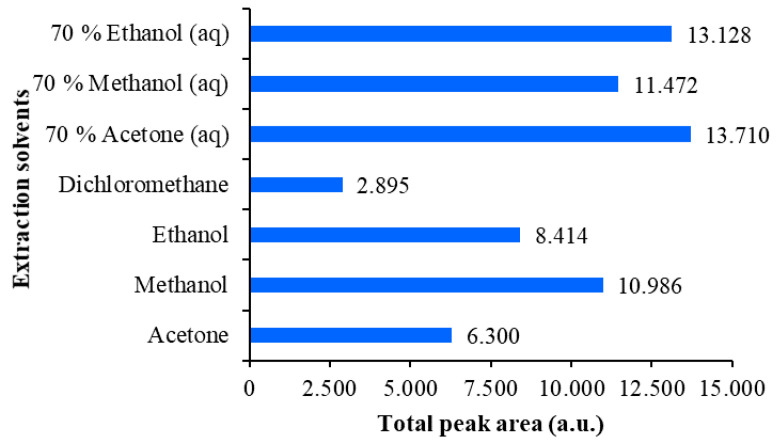
Comparison of the total peak areas obtained from densitograms of Japanese knotweed rhizomes STSs scanned at 442 nm on the HPTLC silica gel plate developed with toluene–acetone-formic acid (3:6:1, *v*/*v*). STSs (2 μL, 50 mg/mL) were prepared with 70% ethanol_(aq)_, ethanol, 70% methanol_(aq)_, methanol, 70% acetone_(aq)_, acetone and dichloromethane.

**Figure 6:**
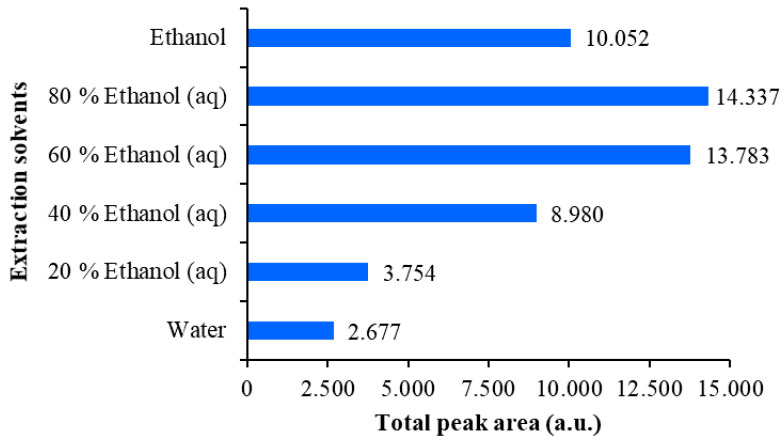
Comparison of the total peak areas obtained from densitograms of Japanese knotweed rhizomes STSs scanned at 442 nm on the HPTLC silica gel plate developed with toluene–acetone-formic acid (3:6:1, *v*/*v*). STSs (2 μL, 50 mg/mL) were prepared with water, 20% ethanol_(aq)_, 40% ethanol_(aq)_, 60% ethanol_(aq)_, 80% ethanol_(aq)_ and ethanol.

The extraction efficiency of all tested extraction solvents was evaluated comparing the total peak areas in the densitograms of STSs prepared with different extraction solvents (Figure 5 and Figure 6). This comparison was performed separately for each HPTLC plate, because peak areas on two different plates cannot directly be compared. On the first HPTLC plate, the highest total peak area was obtained with 70% acetone_(aq)_ and the lowest with dichloromethane (Figure 5). The total peak areas for STSs prepared with other pure organic solvents (acetone, methanol and ethanol) were lower compared to those obtained for STSs prepared with mixtures of organic solvents with water (70% acetone_(aq)_, 70% methanol_(aq)_ and 70% ethanol_(aq)_). On the second HPTLC plate, the highest total peak area was achieved with 80% ethanol_(aq)_ and the lowest with water (Figure 6). Total peak areas increased as the % of ethanol in the extraction solvent was increased from 20% to 80%. However, the total peak area obtained with ethanol was lower compared to that obtained with 60% ethanol_(aq)_. Our results are in agreement with those obtained for the extraction of anthraquinones and other phenolic compounds from Japanese knotweed rhizomes using water and mixtures of water and ethanol (25%, 50%, 75% and 95% ethanol_(aq)_) [24], which showed that 75% ethanol_(aq)_ was the most efficient extraction solvent. Based on our data, 70% acetone_(aq)_ was selected as the extraction solvent, and STS prepared with this solvent was analyzed by HPTLC and HPTLC-MS/MS methods.

### 2.2. HPTLC Analyses

**Figure 7:**
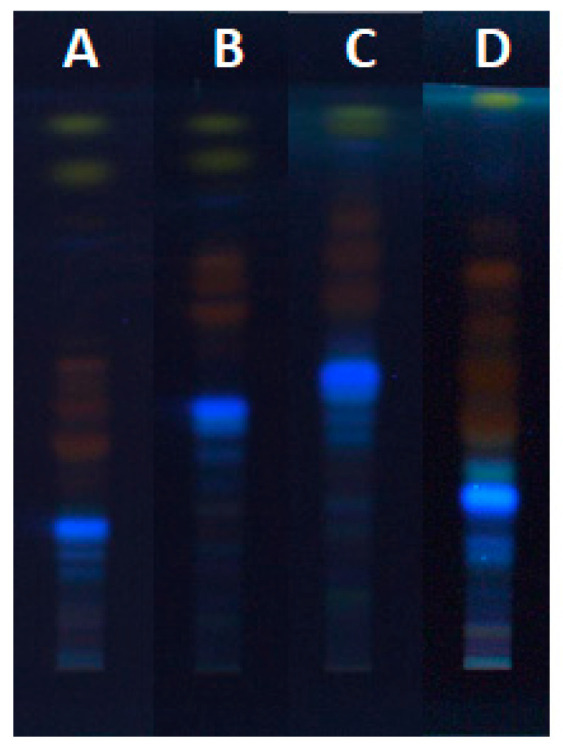
HPTLC chromatograms of Japanese knotweed rhizomes STS (2 μL, 50 mg/mL in 70% acetone(_aq_)) developed up to 9 cm with toluene–acetone–formic acid (6:6:1, *v*/*v*) (**A**), toluene–acetone–formic acid (3:3:1, *v*/*v*) (**B**), toluene–acetone–formic acid (3:6:1, *v*/*v*) (**C**) and dichloromethane–acetone–formic acid (1:1:0.1, *v*/*v*) (**D**) and documented at 366 nm.

Developing solvents with different ratios of toluene–acetone–formic acid (6:6:1, *v*/*v*, 3:6:1, *v*/*v* and 3:3:1, *v*/*v*), as well as dichloromethane–acetone–formic acid (1:1:0.1, *v*/*v*) (Figure 7), were tested as possible developing solvents for the separation of anthraquinones from Japanese knotweed rhizomes STSs in 70% acetone_(aq)_. These solvents were used in our previous study for the separation of proanthocyanidins [12]. In the present study, proanthocyanidins were detected by post-chromatographic derivatization with 4-dimethylaminocinnamaldehyde (DMACA) in the STSs prepared with water, 20% ethanol_(aq)_, 40% ethanol_(aq)_, 60% ethanol_(aq)_, 80% ethanol_(aq)_ and ethanol (Figure 2C).

**Figure 8:**
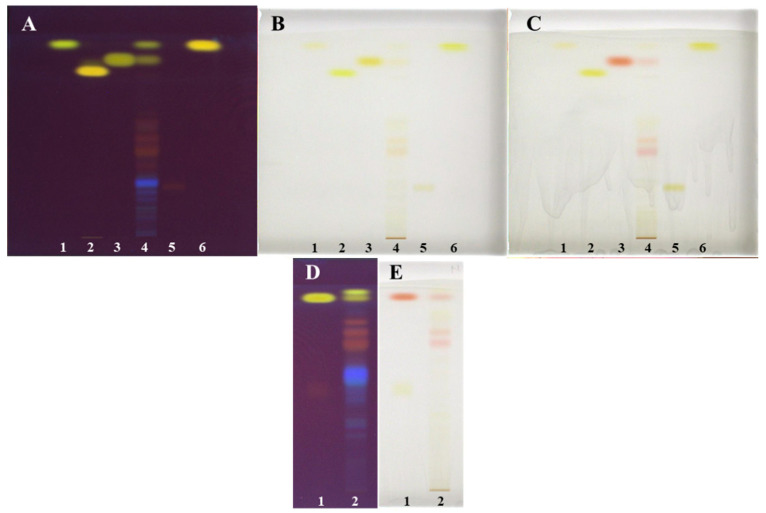
HPTLC chromatograms of standards and Japanese knotweed rhizomes STSs (2 μL, 50 mg/mL) prepared with 70% acetone_(aq)_ (track 4 on the plates (**A**–**C**) and track 2 on the plates (**D**,**E**)). HPTLC silica gel plates were developed with toluene–acetone–formic acid (6:6:1, *v*/*v* (**A**–**C**) or 3:6:1, *v*/*v*; (**D**,**E**)). Documentation was performed at 254 nm after development (**A**,**D**) and at white light after post-chromatographic derivatization with NP reagent (**B**) and 30 min after use of PEGreagent (**C**,**E**). Applications of standards on the plates (**A**–**C**): physcion (1 μg; track 1), aloe-emodin (1 μg; track 2), emodin (1 μg; track 3), aloin A (1 μg; track 5) and chrysophanol (1 μg; track 6). Applications of standards (1 μg; track 1) on the plates: (**D**,**E**) emodin (higher R_F_ and lower R_F_).

As shown in Figure 7, all tested developing solvents enabled separation of non-glycosylated (yellow bands at 366 nm) and glycosylated (orange-brown bands at 366 nm). Only with the developing solvents with different ratios of toluene–acetone–formic acid (6:6:1 (*v*/*v*), Figure 7A, 3:3:1, (*v*/*v*), Figure 7B and 3:6:1, (*v*/*v*), Figure 7C) separation of non-glycosylated anthraquinones was achieved. Among these solvents, the highest resolution between the two yellow bands, later confirmed as emodin and physcion, was achieved with toluene–acetone–formic acid (6:6:1, *v*/*v*) (Figure 7A). Based on the presence of only one yellow band on the plate developed with dichloromethane–acetone–formic acid (1:1:0.1, *v*/*v*), it can be concluded that this developing solvent was not suitable for the separation of non-glycosylated anthraquinones (Figure 7D). The best developing solvents for the separation of glycosylated anthraquinones (orange-brown bands at 366 nm) were dichloromethane–acetone–formic acid (1:1:0.1 (*v*/*v*), Figure 7D) and toluene–acetone–formic acid (6:6:1 (*v*/*v*), Figure 7A). These two solvents enabled separation of six glycosylated anthraquinones, while toluene–acetone–formic acid (3:3:1, *v*/*v*, Figure 7B) and toluene–acetone–formic acid (3:6:1, *v*/*v*, Figure 7C) enabled to separate only four and three glycosylated anthraquinones, respectively. The analysis of STSs (prepared with 70% acetone_(aq)_) together with emodin and aloin A standards on the HPTLC silica gel plates developed with toluene–acetone–formic acid (3:6:1, *v*/*v*) confirmed the presence of emodin by matching the R_F_ values and the band colors of emodin standard and the corresponding band in the STS track after development and after post-chromatographic derivatization with natural product (NP) and polyethylene glycol (PEG) reagents (Figure 8D,E). Colors of both bands changed from yellow after development and NP reagent to red after PEG reagent. Aloin A was not detected in STS. Additional orange-brown bands with the R_F_ values lower than emodin and higher than aloin A were detected but were not identified. Some of these bands become red at white light after the application of PEG reagent (Figure 8E).

**Figure 9:**
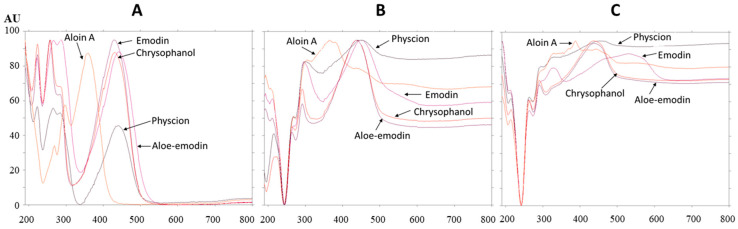
Absorption spectra of standards scanned in situ on the HPTLC silica gel plate developed with toluene–acetone–formic acid (6:6:1, *v*/*v*) after development (**A**), after post-chromatographic derivatization with NP reagent (**B**) and 30 min after use of PEG reagent (**C**). The application of each standard was 1 μg.

Since toluene–acetone–formic acid (6:6:1, *v*/*v*) resulted in the best separation of non-glycosylated and glycosylated anthraquinones among all tested developing solvents, it was chosen for further HPTLC analysis of STS prepared with 70% acetone_(aq)_ on the silica gel plates, together with applied standards of physcion, chrysophanol, aloe-emodin, emodin and aloin A (Figure 8A–C). It is evident that the resolution between the bands of physicon (R_F_ 0.93, track 1, Figure 8A–C) and chrysophanol (R_F_ 0.94, track 6, Figure 8A–C) is not good enough. However, there is a difference in shades of yellow colors of the bands and the shapes of their absorption spectra (Figure 9). The presence of emodin and physcion in STS was confirmed based on matching the R_F_ values and the colors of the bands of the standards (emodin (R_F_ 0.85) and physicon (R_F_ 0.93)) with the corresponding bands in the STS track after development and after post-chromatographic derivatization with NP and PEG reagents (Figure 8A–C). Among the standards, only emodin changed color at white light from yellow to red after using the combination of NP and PEG reagents, while physcion, chrysophanol, aloe-emodin and aloin A remained yellow as they were after the development and after the use of NP reagent (Figure 8A–C). The band of aloin A changed its color at 254 nm and 366 nm from orange-brown after development to intensive light green after the application of NP and PEG reagents (data not shown).

The absorption spectra of all standards were scanned after development (Figure 9A), after post-chromatographic derivatization with NP reagent (Figure 9B) and after the application of PEG reagent (Figure 9C). Bathochromic shifts were observed for the absorption maxima of all standards after post-chromatographic derivatization with NP reagent and, also, after use of PEG reagent (Table 1). These shifts were from 4 nm (aloin A) up to 9 nm (aloe–emodin) after the application of NP reagent. Bathochromic shifts were even more pronounced when the application of NP reagent was followed by PEG reagent. The differences between the absorption maxima of the standards on the same plate after development and after post-chromatographic derivatization (with NP reagent, followed by the application of PEG reagent) were compared. The highest difference (86 nm) was obtained for emodin.

**Figure 10:**
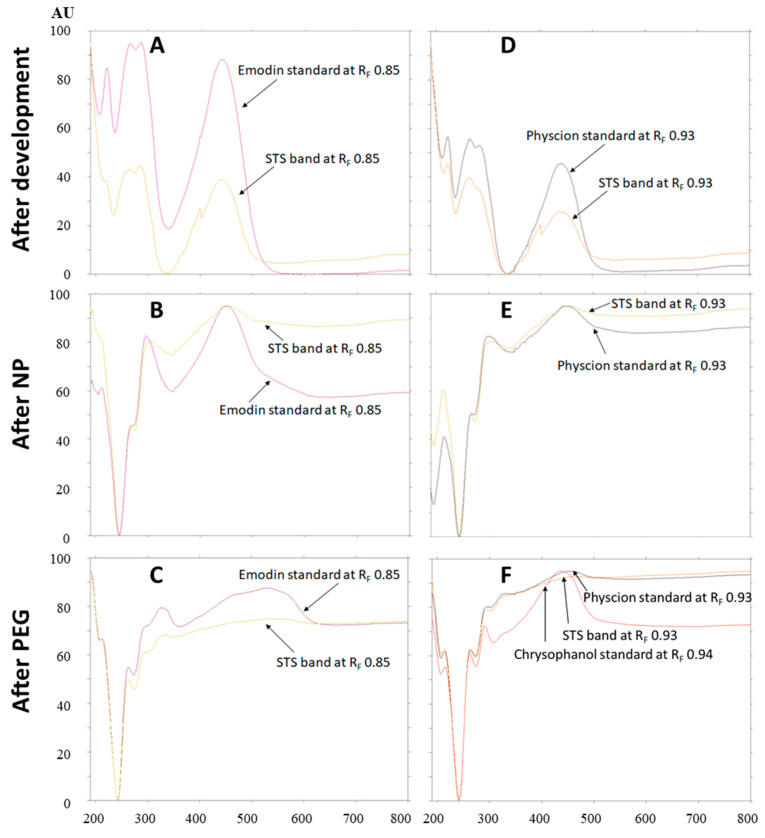
Absorption spectra of emodin (at R_F_ 0.85; **A**–**C**) and physcion (at R_F_ 0.93; **D**–**F**) standards and compounds in the bands at the same R_F_s (**A**–**C**: R_F_ 0.85 and **D**–**F**: R_F_ 0.93) in the track of STS (2 μL, 50 mg/mL, 70% acetone_(aq)_). Spectra were scanned in situ on the HPTLC silica gel plate developed with toluene–acetone–formic acid (6:6:1, *v*/*v*) after development, after post-chromatographic derivatization with NP reagent and 30 min after use of PEG reagent. Additional absorption spectrum of chrysophanol scanned after post-chromatographic derivatization with NP reagent and 30 min is presented in (**F**).

Absorption spectra of emodin (R_F_ 0.85) and physcion (R_F_ 0.93) standards and the absorption spectra of compounds in the corresponding bands in the STS track were scanned in situ in the range of 190–800 nm. Comparison of the absorption spectra of emodin (R_F_ 0.85) and compounds in the corresponding STS band with the same R_F_, scanned after development (Figure 10A), after post-chromatographic derivatization with NP reagent (Figure 10B) and after the application of PEG reagent (Figure 10C) confirmed that the spectra matched. This was additional proof of the presence of emodin in the STS. Comparison of the absorption spectra of physcion (R_F_ 0.93) and compounds in the corresponding STS band with the same R_F_ scanned after development (Figure 10D), after post-chromatographic derivatization with NP reagent (Figure 10E) and after the application of PEG reagent (Figure 10F) confirmed that the spectra matched. The possible presence of chrysophanol (R_F_ 0.94), which was not separated from physcion (R_F_ 0.93), was rejected. The rejection was based on the comparison of the absorption spectra of chrysophanol, physcion and compounds in the corresponding STS band with the same R_F_ (Figure 10F). Chrysophanol has a different shape of absorption spectrum and also different absorption maxima after development, after post-chromatographic derivatization and after use of PEG reagent (Table 1).

Other compounds present in orange-brown bands (at 366 nm; Figure 8A,D) could not be identified by HPTLC analyses due to the lack of appropriate standards. However, they were tentatively identified as glycosylated anthraquinones by HPTLC-MS/MS analyses (Section 2.3) when their fragmentation patterns were compared with those reported in the literature [6,20].

### 2.3. HPTLC-MS/MS Analyses

**Figure 11:**
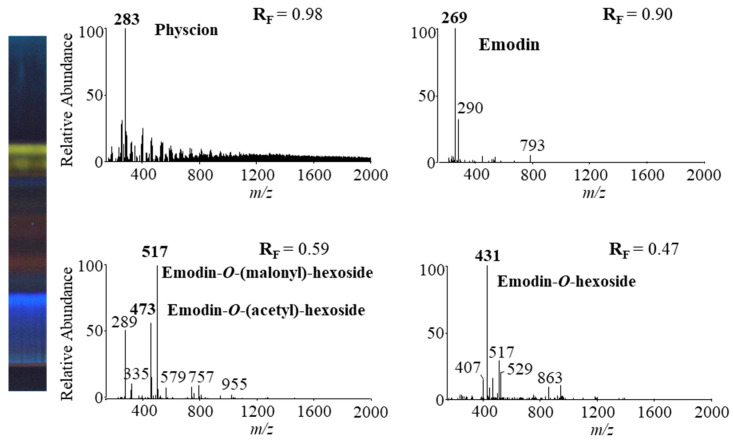
MS spectra of anthraquinones (bold, *m/z*) obtained from Japanese knotweed rhizomes STS from a twice predeveloped HPTLC silica gel plate developed up to 7 cm with toluene–acetone–formic acid (6:6:1, *v*/*v*) as the developing solvent and documented at 366 nm.

Four HPTLC-MS methods (Table 2) were used to analyze anthraquinones from STS of Japanese knotweed rhizomes to obtain MS and MS^n^ spectra. HPTLC silica gel plates were twice predeveloped (up to the top) before development up to 7 cm with the following developing solvents: toluene–acetone–formic acid (3:6:1 (*v*/*v*), 6:6:1 (*v*/*v*) and 3:3:1 (*v*/*v*)) and dichloromethane–acetone–formic acid (1:1:0.1, *v*/*v*). MS spectra obtained by HPTLC-MS analyses using toluene–acetone–formic acid (6:6:1, *v*/*v*) as the developing solvent are presented in Figure 11.

The identification of emodin and physcion in Japanese knotweed rhizomes STSs was confirmed by comparing the absorption spectra of yellow bands (Figure 10) and the fragmentation patterns (at R_F_s corresponding to R_F_s for physcion and emodin standards) (Table 2) with absorption spectra and fragmentation patterns of physcion and emodin standards. Other compounds present in orange-brown bands were tentatively identified as glycosylated anthraquinones by comparison of the fragmentation patterns (Table 2) with those reported in the literature. The MS^2^ spectrum of the signal at *m*/*z* 431 gave a base ion at *m*/*z* 269 with a neutral loss of 162 Da, and the MS^3^ spectrum gave product ions at *m*/*z* 225 and *m*/*z* 241, which were identical to emodin standard. This indicated that signal *m*/*z* 431 could be emodin hexoside. Emodin-1-*O*-glucoside [2,3,6], emodin-6-*O*-glucoside [6] and emodin-8-*O*-glucoside [2,3,6] were previously identified in Japanese knotweed rhizomes. The MS^2^ fragmentation pattern of a base peak at *m*/*z* 431 was similar to the fragmentation pattern (product ions at *m*/*z* 269 and *m*/*z* 311) of emodin-8-*O*-glucoside [2,6] and the MS^3^ fragmentation pattern was typical for emodin reference standard. Therefore, the signal at *m*/*z* 431 was tentatively assigned to [M−H]^−^ of emodin-8-*O*-hexoside.

The MS^2^ and MS^3^ fragmentation patterns of the signal at *m*/*z* 517 were similar to the fragmentation pattern of emodin-8-(6′ malonyl)-glucoside [20], which were reported to be present in Japanese knotweed rhizomes [2,3,6]. Therefore, the signal at *m*/*z* 517 was tentatively identified as emodin–malonyl–hexoside. Another signal at *m*/*z* 473 was obtained at the same R_F_ value using all HPTLC-MS/MS methods. The MS^2^ spectrum of a signal at *m*/*z* 431 gave a base ion at *m*/*z* 269. A loss of 204 Da indicated acetyl and glucosyl residues. MS^3^ spectrum of the signal at *m*/*z* 473 gave product ions at *m*/*z* 225 and *m*/*z* 241, which correspond to emodin. Therefore, the signal at *m*/*z* 473 was tentatively assigned as emodin–acetyl–glucoside, which was previously identified in Japanese knotweed rhizomes [6].

HPTLC-MS/MS methods using different ratios of toluene–acetone–formic acid (3:6:1, *v*/*v*, 6:6:1, *v*/*v* and 3:3:1, *v*/*v*) and dichloromethane–acetone–formic acid (1:1:0.1, *v*/*v*) as the developing solvents enabled the detection of emodin and its three glycosylated analogs (Table 3). The method with toluene–acetone–formic acid (6:6:1, *v*/*v*) additionally enabled the detection of physcion.

## 3. Materials and Methods

### 3.1. Chemicals

All chemicals used in this study were at least of analytical grade. Toluene, dichloromethane, ethyl acetate hydrochloric acid (37%), formic acid and 4-dimethylaminocinnamaldehyde (DMACA) were purchased from Merck (Darmstadt, Germany). Diphenylboric acid 2-aminoethyl ester (natural product reagent, NP), ethanol (absolute), acetone and HPLC grade methanol and acetonitrile were from Sigma-Aldrich (St. Louis, MO, USA). LC-MS grade methanol and acetonitrile were from Fluka (Buchs, Switzerland). Polyethylene glycol (PEG) 4000 was from Fluka Chemie (Buchs, Switzerland). Bidistilled water was also used.

Standards of physcion, chrysophanol, aloe-emodin, emodin and aloin A were obtained from Extrasynthesè S.A. (Genay, France).

### 3.2. Preparation of Standard Solutions

Standard solutions (0.2 mg/mL) were separately prepared in methanol and were stored in amber glass storage vials at −80 °C.

### 3.3. Plant Material and Preparation of Sample Test Solutions (STSs)

Rhizomes (a subterranean stem) of Japanese knotweed (*Fallopia japonica* Houtt.) were collected at Ljubljanica riverside in Ljubljana (Vrhovci, by bridge over Mali graben, N 46°02′33.9″; E 14°27′00.9″ [34]) in August, 2019. The voucher specimen is deposited in the Herbarium LJU (LJU10143477).

The rhizomes were washed with tap water, dried on air, cut into smaller pieces, frozen with liquid nitrogen and lyophilized (Micro Modulyo, IMAEdwards, Bologna, Italy) for 48 h at −50 °C. Freeze-dried rhizomes were again frozen with liquid nitrogen, crushed and pulverized by Mikro-Dismembrator S (Sartorius, Göttingen, Germany) at a frequency of 1700 min^−1^ for 1 min.

Powdered lyophilized plant material (100 mg) was dispersed in 4 mL of extraction solvent. Water, 20% ethanol_(aq)_, 40% ethanol_(aq)_, 60% ethanol_(aq)_, 70% ethanol_(aq)_, 80% ethanol_(aq)_, ethanol, 70% methanol_(aq)_, methanol, 70% acetone_(aq)_, acetone and dichloromethane were used as extraction solvents. Suspensions were vortexed (2 min at 2800 rpm; IKA lab dancer, Sigma-Aldrich) and centrifuged at 6700× *g*. Supernatants were filtered through a 0.45-µm polyvinylidene fluoride (PVDF) membrane filter (Millipore, Billerica, MA, USA). The obtained sample test solutions (STSs) (50 mg/mL) were stored in amber glass storage vials at −80 °C and were used undiluted for the HPTLC and HPTLC-MS analyses.

### 3.4. HPTLC Analyses

HPTLC analyses were performed on 20 cm × 10 cm glass-backed HPTLC silica gel (Merck, Art. No. 1.05641, Darmstadt, Germany). Standard solutions (1 µg) and STSs, which were prepared in different extraction solvents (2 µL), were applied on the un-predeveloped plates by an automatic TLC Sampler 4 (Camag, Muttenz, Switzerland). The plates were developed up to 9 cm using different ratios of toluene–acetone–formic acid (3:6:1, *v*/*v*, 6:6:1, *v*/*v* and 3:3:1, *v*/*v*) and dichloromethane–acetone–formic acid (1:1:0.1, *v*/*v*) as the developing solvents. The developing solvent (10 mL) was added only in one trough of an unsaturated twin-trough chamber (Camag) for 20 cm × 10 cm plates. Only 5 mL of the developing solvent was used in case of 10 × 10 cm or smaller plates, which were developed in a twin-trough chamber (Camag) for 10 cm × 10 cm plates. The developed plates were dried in a stream of warm air for 3 min.

Post-chromatographic derivatization was performed by heating the plates on a TLC plate heater III (Camag) at 110 °C (3 min), which was immediately followed by dipping the plate for 1 s in the natural product reagent (NP reagent), prepared by dissolving 1 g of NP in 200 mL of ethyl acetate [35]. After drying in a stream of warm air (hair dryer) for 2 min, followed by cooling in the air for 5 min, the plates were dipped in PEG 4000 reagent, prepared by dissolving 10 g of PEG 4000 [35] in 200 mL of dichloromethane. The plates were again dried in a stream of warm air (hair dryer) for 2 min. Additional post-chromatographic derivatization was used to detect proanthocyanidins. In this case, plates were dipped for 1 s in DMACA dipping detection reagent prepared by dissolving 60 mg of DMACA in 13 mL of concentrated hydrochloric acid, which was made up to 200 mL with ethanol [36]. Dipping was followed by drying for 2 min in a stream of warm air. NP reagent, PEG reagent and DMACA reagent were protected from light and stored at 5 °C.

The Camag Digistore 2 Documentation system in conjunction with Reprostar 3 was used to document the images of the chromatographic plates at 254 nm, 366 nm and white light illumination. The plates were documented: (i) immediately after development, (ii) immediately after post-chromatographic derivatization with NP reagent, (iii) immediately after the enhancement and stabilization of fluorescent zones with PEG reagent and (iv) 30 min after the enhancement and stabilization of fluorescent zones with PEG reagent. When DMACA reagent was applied for post-chromatographic derivatization, the images were documented only at white light illumination immediately and 10 min after derivatization. After documentation the developed plates were scanned by the slit-scanning densitometer TLC Scanner 3 (Camag) set in absorption/reflectance mode at 442 nm. The slit length was 6 mm, the slit width 0.30 mm and the scanning speed 20 mm s^−1^. The absorption spectra (from 190 nm to 800 nm) were scanned in situ before and after post-chromatographic derivatization with NP reagent and also after application of PEG reagent. Both instruments were controlled by winCATS software (Version 1.4.9.2001).

### 3.5. HPTLC-MS/MS Analyses

The HPTLC silica gel plates were firstly predeveloped with methanol–formic acid (10:1, *v*/*v*) and, secondly, with acetonitrile–methanol (2:1, *v*/*v*)) up to the top and dried for 30 min at 100 °C. Twice predeveloped plates (cut to 5 cm × 10 cm) were used for the application of 20 µL of STS in 70% acetone_(aq)_ as 24 mm band, 10 mm from the bottom edge, 20 mm from the left edge and developed up to 7 cm for the HPTLC-MS/MS analyses of anthraquinones. Toluene–acetone–formic acid (3:6:1 (*v*/*v*), 6:6:1 (*v*/*v*) and 3:3:1 (*v*/*v*)) and dichloromethane–acetone–formic acid (1:1:0.1, *v*/*v*) were used as the developing solvents.

The yellow bands that appeared on the developed plates were used for positioning the oval elution head (4 mm × 2 mm) of the TLC-MS interface (Camag) and were eluted and transferred into a LCQ ion trap system (Thermo Finnigan, San Jose, CA, USA). For evaluation of the collected data Xcalibur 1.3 software was used. HPTLC–MS analyses were performed according to [12,13]. Acetonitrile–methanol (2:1, *v*/*v*) was used as an eluent at 0.2 mL min^−1^ flow rate. A C18 guard column (4 mm × 3 mm ID, Phenomenex, Torrance, CA, USA) was mounted between the TLC–MS interface and MS ion source. Electrospray ionization (ESI) in negative ion mode was used to acquire mass spectra from *m*/*z* 150–2000 scan range in 1 min. The spray voltage was set to 4 kV, capillary temperature to 200 °C, capillary voltage to −38.8 V, tube lens offset to −5, flow rate sheath gas to 95 a.u. (arbitrary units) and flow rate auxiliary gas to 14 a.u. Fragmentation of the parent ion was performed at 45% collision energy.

## 4. Conclusions

Twelve extraction solvents (water, 20% ethanol_(aq)_, 40% ethanol_(aq)_, 60% ethanol_(aq)_, 70% ethanol_(aq)_, 80% ethanol_(aq)_, ethanol, 70% methanol_(aq)_, methanol, 70% acetone_(aq)_, acetone and dichloromethane) were used for the extraction of anthraquinones (yellow dyes) from Japanese knotweed rhizomes. The obtained sample test solutions (STSs) were analyzed by the HPTLC method on HPTLC silica gel plates developed with toluene–acetone–formic acid (3:6:1, *v*/*v*). Qualitative densitometric profiles scanned at 442 nm (absorption maximum for emodin) for STSs prepared in all the solvents except dichloromethane were identical and included anthraquinone aglycones and glycosylated anthraquinones). The most selective extraction of anthraquinone aglycones emodin and physcion was achieved with dichloromethane. Extraction efficiency, evaluated by comparison of the total peak areas of the densitograms of STSs prepared with all solvents, was the highest with 70% acetone_(aq)_. Therefore, only STS prepared with this solvent was used for further selection of developing solvent for HPTLC and HPTLC-MS analyses.

HPTLC silica gel plates in combination with four new developing solvents were proposed for analyses of anthraquinones by HPTLC and HPTLC-MS. All developing solvents with different ratios of toluene–acetone–formic acid (6:6:1 (*v*/*v*), 3:6:1 (*v*/*v*) and 3:3:1 (*v*/*v*)) and dichloromethane–acetone–formic acid (1:1:0.1, *v*/*v*) enabled the separation of non-glycosylated and glycosylated anthraquinones present in STS prepared with 70% acetone_(aq)_. However, the separation of non-glycosylated was achieved only with the developing solvents with different ratios of toluene–acetone–formic acid (6:6:1 (*v*/*v*), 3:6:1 (*v*/*v*) and 3:3:1 (*v*/*v*)). The best resolution between non-glycosylated anthraquinones emodin and physcion was achieved with toluene–acetone–formic acid 6:6:1 (*v*/*v*). The best separation of glycosylated anthraquinones was achieved with toluene–acetone–formic acid (6:6:1, *v*/*v*) and dichloromethane–acetone–formic acid (1:1:0.1, *v*/*v*). Changes of the shapes and the absorption maxima (bathochromic shifts) in the absorption spectra of the standards (physcion, chrysophanol, aloe–emodin, emodin and aloin A) scanned in situ on the HPTLC plate after development, after post-chromatographic derivatization with NP reagent and after the application of PEG reagent were observed. These changes were used as additional proof for the presence of emodin and physcion (almost the same R_F_ but different absorption spectra than chrysophanol) in STS prepared with 70% acetone_(aq)_ analyzed on the same plate as the standards. All four HPTLC-MS/MS methods using different ratios of toluene–acetone–formic acid (3:6:1 (*v*/*v*), 6:6:1 (*v*/*v*) and 3:3:1 (*v*/*v*)) and dichloromethane–acetone–formic acid (1:1:0.1, *v*/*v*) as the developing solvents enabled the identification of emodin and tentative identification of its three glycosylated analogs (emodin-8-*O*-hexoside, emodin-*O*-acetyl-hexoside and emodin-*O*-malonyl-hexoside). Additionally, the HPTLC-MS/MS method with toluene–acetone–formic acid (6:6:1, *v*/*v*) enabled the identification of physcion. The identification of emodin and physcion in Japanese knotweed rhizomes STS was confirmed by comparing the absorption spectra and the fragmentation patterns (at R_F_s corresponding to R_F_s for emodin and physcion standards) with fragmentation patterns of physcion and emodin standards.

## Figures and Tables

**Table 1:** Absorption maxima of standards determined by densitometric scanning of the HPTLC silica gel plate developed with toluene–acetone–formic acid (3:6:1, *v*/*v*). Spectra were scanned after development, after post-chromatographic derivatization with NP reagent and 30 min after the application of PEGreagent.

	Absorption Maxima of Standards (nm)		
Compounds	After Development	After NP	30 min after PEG
Physcion	442	449	454
Aloe-emodin	428	437	438
Emodin	442	449	528
Aloin A	360	364	387
Chrysophanol	431	437	437

**Table 2:** Anthraquinones tentatively identified in Japanese knotweed rhizomes by HPTLC- MS/MS.

Compound	(-)ESI-MS *m*/*z*	(-)ESI-MS^n^ **m/*z*	Ref.
Emodin ^a^	269	MS^2^ [269]: 225, 241	
		MS^3^ [269→225]: 181, 210, 197, 207	
Physcion ^a^	283	MS^2^ [283]: 240, 268	
		MS^3^ [283→240]: 212	
Emodin-8-*O*-hexoside	431	MS^2^ [431]: 269, 311	[20]
		MS^3^ [431→269]: 225, 241	
Emodin-*O*-acetyl-hexoside	473	MS^2^ [473]: 269, 311	[6]
		MS^3^ [473→269]: 225, 241	
Emodin-*O*-malonyl-hexoside		MS^2^ [517]: 473	[20]
		MS^3^ [517→473]: 269, 311	
		MS4 [517→269]: 225, 241	

^a^ Confirmed with a reference standard.

**Table 3:** R_F_ values of anthraquinones identified by HPTLC-MS analyses in Japanese knotweed rhizomes sample test solutions (STSs) (prepared with 70% acetone_(aq)_) on HPTLC silica gel plates developed with different developing solvents.

Compound	*m*/*z*	Developing Solvent			
		DS1	DS2	DS3	DS4
		R_F_			
Emodin	269	0.90	0.87	0.97	0.99
Physcion	283	0.98	/	/	/
Emodin-*O*-hexoside	431	0.47	0.62	0.68	0.47
Emodin-*O*-(acetyl)-hexoside	473	0.59	0.74	0.81	0.81
Emodin-*O*-(malonyl)-hexoside	517	0.59	0.74	0.81	0.81

DS1: toluene–acetone–formic acid (6:6:1, *v*/*v*). DS2: toluene–acetone–formic acid (3:3:1, *v*/*v*). DS3: toluene–acetone–formic acid (3:6:1, *v*/*v*). DS4: dichloromethane–acetone–formic acid (1:1:0.1, *v*/*v*).
